# Honesty Is the Best Policy

**Published:** 1871-11

**Authors:** 


### "HONESTY IS THE BEST POLICY."

That "honesty is the best policy," is a maxim so often quoted that its very triteness has deprived it of much of its force. Indeed many are inclined to doubt its truth, nor are their doubts wholly without reason. Villany often has an appearance of success. We not unfrequently see the base and unscrupulous have the rarest gifts of fortune heaped upon them in profusion, while the really deserving seem hard beset by the rude blasts of adversity. Especially in these days when profligacy has become the prevailing fashion, and rascality, is a sure passport to wealth and power, is there cause to doubt the truth of this maxim Practices which prostitute every noble and generous quality are now the means by which colossal fortunes are amassed and places of distinction are attained. Knavery wins all the high figures in the lottery, while to honesty is left little more than blanks. Under circumstances like these, it would seem vastly wise to be wicked.

But it would appear so only to an imperfect observer. The outward seeming is not always the truth, and to him who looks beneath the surface it will be evident, beyond question, that "honesty is the best policy." That which seems to be success may fall much short of being so in reality. One may rise to position of distinction and influence, may heap up much wealth, and gain a large share of a certain kind of fame, and yet his life be to his own consciousness, a miserable failure. The arts and means to which he has resorted, to attain these ends, has lost him his own self-respect and the respect of his fellow-men; and without these, all the external marks of success are but as Dead Sea fruit. Nothing won by dishonesty and trickery can give any very real or lasting happiness.

But apart from the reproofs of conscience, which one must endure who *has* given himself up to unholy ambition or avarice, there must be a continual dread of that day of reckoning which generally overtakes the vicious, sooner or later, and which, like the sword of Damocles, destroys the pleasure of many a joyous scene. One must doubt the performance of fortune or fame built upon wrong doing. Revelations may burst forth when least expected, and the avenging hand may smite with destruction when but a moment before, all seemed calm and secure. How many instances have we seen of this in the past few years. How many thrones that were established upon usurpation and tyranny have we seen suddenly swept away. There are many in our Heaven-born profession who have climbed to high places by fraud and treachery, whose coming fate will teach the same great lesson. Every one who is tempted by the prospect of gain to enter upon a course of wrong doing, will find perhaps when too late, that it would have been better to have done right.

Bacon has asserted that the maxim which we have quoted seldom if ever influences the conduct of any one. "The truly honest man," says he, "is always in advance of it, and the rascal is behind it." True, but there are very many whom we can class neither as honest men nor rascals. Between those who adhere to principle regardless of expediency, and those who are utter villains there lies a large class who are neither one nor the other. These may be impelled to do right by having it impressed upon them that it is best to do so, and we think we have the highest sanction for making such appeal. "What will it profit a man if he gain the whole world and lose his own soul?" is the fearfully momentous question propounded by the Saviour to this class. Evidently He was not addressing men who gave principle the preference over all things else. He was appealing to men whom the love of power and wealth might lead to sin, and depicting most forcibly the utter worthlessness of these things when compared with that purity of faith and practice which insures the salvation of the soul.

If He, the Great Teacher, sought to influence men by a motive like this, surely we are authorized to encourage them to right-doing because to do right is best.

But here let them avoid the error of supposing that which may look and seem right, is best. There can be but one rule on this subject. We *must obey implicitly the decisions of conscience*, sustained by correct principle and law. We must not allow the slightest "departure" from its dictates when thus formed and supported. If we begin to say unto our conscience "pardon us in this thing," or pardon us in that, permit us to ignore principle and law this time, we will soon get to be as if we had no conscience at all, and may sink into the most degrading vices. We do not put this matter too strongly. We must adhere to principle and law, not only in order to maintain a proper degree of moral dignity, but even to be safe from downright villainy. Intellect, however cultivated it may be, is no safe guide in moral questions. Its sophistries are the most wonderful and unaccountable among all the vagaries of human nature. When men begin to *reason* they may easily persuade themselves that they may *lie*, *steal* and *murder*, not only without wrong, but with the most benevolent intentions. The character of Eugene Aram is no creation of the poet's brain. It is a faithful picture of what any man will become who dethrones conscience and establishes *reason* as his moral guide. Reason is unfit for the post. She will soon resign herself to the rule of the passions, and give her sanction to the worst crimes that have ever disgraced humanity.

The most mischievous men who have lived upon the earth have been those who perhaps designed no evil, but who sought to do in some of the tortuous courses which a sophistical intellect rather than the voice of conscience, suggested.

The wanderer upon the ocean seeks among the luminaries of Heaven some beacon by which to direct his way. Many are shining there, but all save one will lead him astray should he select them for his guides, for they are moving as well as his. But there beams the polar star, not so brilliantly indeed as many others, but always in the right place. Whoever directs his course by it will be sure not to go wrong. Truth is the polar star of the moral heavens. Its light never leads astray. Others may for a time be more conspicuous, but it never flickers and never grows dim. He who follows this guide need never fear evil, for his path is the pathway of honor and right. Should he abandon this, there is nothing to which he can look with an eye of faith. All else is fleeting, changeable, uncertain, and those who pursue such must be unstable and uncertain, too. The politician who abandons truth and principle to follow after expediency for *votes*, if he succeeds will have to make "a new departure" every few days. The lawyer who strives for what may seem his own success rather than for what is truly right, prostitutes himself and his profession, and becomes the basest of men. The physician who betrays the cause of scientific truth for some petty personal advantage is unworthy to have his name enrolled in that noble profession. The minister of Religion who, in order to win some popularity or worldly honor compromises the truth, deserves a degree of condemnation which no language can express. In short, we cannot denounce too harshly those who, for love of money or fame, disregard principle and follow after that which may seem expedient. Such persons suffer indeed no slight punishment in the degredation of their moral natures, but they deserve likewise the contempt of all whose good opinion is in the least degree valuable.

No, it does not always seem the best to do right, and it is often very hard to do so. The path of duty frequently seems very hard and rugged, while beside it lie pleasant walks, beneath embowering shades resonant with sweet sounds and fragrant with delicious perfumes, But beware how you leave the dusty highway to stray therein. Beneath the flowers blooming so gayly are hidden gins and snares innumerable, into which, if the unwary but should fall, they will rise no more. The way of truth seems not so pleasant; but the end is more happy. The Great Teacher, when pledging

His disciples to faith in the life-giving truth which He was about to impart, plainly forwarned them that it was to no life of ease and selfish indulgence that they were being called. He notified them that they would have to encounter ridicule and persecution even unto death. In return for all this, He promised the sweet approval of their consciences in this life, and joys indescribin the life to come. What higher reward could He offer? The approval of one's own conscience—a mind conscious within itself of rectitude! Why for this one might well face ridicule, persecution and death. It constitutes the highest bliss that can be known on earth, and we conceive is not least among the joys of Heaven.

